# The Hypophosphate in Cholera Infantum

**Published:** 1871-10

**Authors:** T. Taylor

**Affiliations:** Fishersville, Ky.


THE GEORGIA

### MEDICAL COMPANION:

A MONTHLY ADVISER.

VOL. I. ATLANTA, GA., OCTOBER, 1871. NO. 10

### PART I.

### Original Communications and Special Selections.

### THE HYPOPHOSPHITES IN CHOLERA INFANTUM.

BY T. TAYLOR, M. D., FISHERSVILLE, KY.

The great hopes that were raised, by the publication of Dr. Churchill's observations on the use of the Hypophosphites in Phthisis—and the consequent disappointment from a failure to verify his opinions, has very much detracted, we think, from the value of these remedies in other morbid conditions of the system, and caused them to fall to a point among remedial agents, far below that to which they are fairly and justly entitled.

The experiments of Dr. De Ricci, of Dublin, and Dr. Purdon, of Belfast, abundantly show the value of these remedies in all kinds of diseases caused by fermentation of poisons in the blood, such as typhus, typhoid, scarlatina, etc., and prove that they have a power of arresting this action of fermentation similar to sulphurous acid in vinous fermentation.

From a notice of Purdon's experiments, particularly in Remittent fever, I was induced to try them in Cholera Infantum, and in some cases of gastric irritation, which had resisted the usual remedies, and in every instance, with the happiest results.

My observations covered about twenty cases of Cholera Infantum; five cases of Chronic Gastric Vomiting, and two cases of Tabes Mesenterica, and of the entire number, not one resulted fatally. I think I am safe in saying that some of these cases, at least, would not have recovered under the ordinary methods of treatment. From this number I select three or four as samples of my method of treatment with the Hypophosphites, premising, however, that after treating a few of the first in the usual manner, the Hypophosphites were commenced at once. I used the formula recommended by Dr. Purdon, as suitable for an infant, the dose being increased according to age:

| | | | |
|---|---|---|---|
| ℞.—Hypohos. Sodæ | - . - | | grs. vi. |
| Hyphos. Calcis, | | | |
| Hypohos. Potass | - - | aa | grs. iv. |
| Glycerine | - - - - - | | dr ij. |
| Aqua Des | - - - - | | oz. j.—M. |

Dose—40 drops 3 times a day for a child one year old, in a little water or milk. Sometimes I found it necessary to repeat the dose every three or four hours.

In all these cases it is proper to say, where it was indicated, the billiary secretion was improved by the administration of small doses of calomel before beginning, and sometimes during the use of the Hypohosphites.

Cholera Infantum.—J. S——, aged fifteen months. Diarrhœa with stools of a most offensive odor, and very frequent. Abdomen hot and distended—extremely fretful and pevish—stomach very irritable, ejecting the milk and water within a few minutes after swallowing. Gums considerably swollen, which were lanced but without mitigating the symptoms. He first had powders of bismuth sub. nit. doveri pul. and creta prepp., and afterwards calomel, plumbi act. and opium every two or three hours with quinia sul. and potass bicar., three times a day. The diet was milk and lime-water. No improvement, and on the third day he had two or three slight convulsions. All the unfavorable symptoms, if possible, were aggravated, the pupils dilated and the child apparently unconscious. At this stage, the hypophosphites were commenced, with the bromide of potassium. In four hours after taking the first dose he dropped off into a calm sleep. The treatment was continued with the addition of tonic doses of quinine, and the improvement was steady and uninterrupted. In this case I was impressed at the time, with the idea that the bromide was the agent in inducing sleep, but subsequent observations prove that a quiet rest almost invariably follows the use of the hypophosphites.

Cholera Infantum.—H. G——, aged two years. Symptoms; alvine evacuations thin, dark-colored, offensive and very frequent. Abdomen, tympantic; much thirst; continual vomiting; pupils dilated; skin hot and dry. The usual treatment was instituted, with tepid bath night and morning and friction over the body. Rich milk with lime-water and the occasional use of arrow-root was the diet, but in spite of all, he grew rapidly worse. The hypophosphites was then commenced, and in two hours, as in the other case, he dropped into a sleep, from which he awoke very much better. The treatment was continued and his convalescence rapid.

Tabes Mesenterica.—W. L——, aged four years. He presented the appearances of a strumous diathesis. Thorax was found to be healthy. The abdomen was hard to the touch, the stools of a whitish, clayey color; appetite capricious; the tongue alternately clean for three or four days, then foul. The parents informed me that "he had been gradually wasting away" for three or four months. This case was doubtless one of the most unpromising I have ever seen. He was at once put upon the use of the hypophosphites with but little hope that any good would result, but to my astonishment a marked improvement was visible after a few days, and this continued until he was in his usual health.

But it is useless to multiply cases, as these will serve to direct the attention of the profession to these remedies in this class of diseases. Indeed, so constant have been the effects of these remedies in my hands, that I feel safe in making a favorable prognosis, no matter how virulent the attack.

It is difficult to understand, with our present limited knowledge of these medicines, how they act. That they have obvious effects on the system, outside of the mere arrest of the action of fermentation in the stomach, I think will be conceded by any one who will take the trouble to note closely their action.

Dr. Churchill's opinion that they "increase the nervous force, and are most powerful hæmatogens, possessing all the therapeutic properties of phosphorous," is abundantly corroborated by my observations. But the influence they exert to check any tendency to diarrhœa and to increase the appetite, as well as their power to produce cheerfulness in children, who have long been fretful and peevish, is not so well understood.

The attention of the profession is called to these remedies with the hope that further observation will enable the medical man to avail himself of the aid of a powerful auxiliary in the treatment of one of our most common and fatal forms of disease.

